# Proteomics: Analysis of Spectral Data

**Published:** 2007-02-24

**Authors:** Harry B Burke

**Affiliations:** Associate Professor of Medicine, Biochemistry and Molecular Biology, Director of Bioinformatics and Biostatistics, McCormick Genomics Center, George Washington University School of Medicine

**Keywords:** proteomics, SELDI-TOF-MS, MALDI, spectra, protein profile, bioinformatics, biostatistics, prediction, top-down processing, bottom-up processing

## Abstract

The goal of disease-related proteogenomic research is a complete description of the unfolding of the disease process from its origin to its cure. With a properly selected patient cohort and correctly collected, processed, analyzed data, large scale proteomic spectra may be able to provide much of the information necessary for achieving this goal. Protein spectra, which are one way of representing protein expression, can be extremely useful clinically since they can be generated from blood rather than from diseased tissue. At the same time, the analysis of circulating proteins in blood presents unique challenges because of their heterogeneity, blood contains a large number of different abundance proteins generated by tissues throughout the body. Another challenge is that protein spectra are massively parallel information. One can choose to perform top-down analysis, where the entire spectra is examined and candidate peaks are selected for further assessment. Or one can choose a bottom-up analysis, where, via hypothesis testing, individual proteins are identified in the spectra and related to the disease process. Each approach has advantages and disadvantages that must be understood if protein spectral data are to be properly analyzed. With either approach, several levels of information must be integrated into a predictive model. This model will allow us to detect disease and it will allow us to discover therapeutic interventions that reduce the risk of disease in at-risk individuals and effectively treat newly diagnosed disease.

## Introduction

Proteins are required for the normal functioning of the body, their dysregulation may be the cause of disease, and they may be the targets of treatment. They are complex three dimensional biochemical structures that can exist in several forms owing to post-translational modification and other processes. Proteins have multiple actions. Its action at any moment in time depends on its structure, its interactions with other proteins, and its biochemical milieu. Proteins tend to exist in small qualities and they usually do not act by mass effect, in other words, the amount of protein is usually not the determinant of its activity. Proteins are intracellular, intercellular, and they circulate in the blood. Most serum proteins are bound; small quantities circulate in free form in the blood. A few proteins exist in high abundance in the serum, but the great majority are in low abundance. Most proteins have not yet been identified. We do not need proteomics to identify the high abundance proteins in the serum, rather, we need it to discover new, low abundance proteins.

Proteins bind to, and interact with, other proteins as complexes. Therefore, proteins cannot be analyzed in isolation from other proteins. The high abundance proteins must be assessed with the low abundance proteins that are bound to them and the high abundance proteins must then be removed from the analysis. Proteomic representation technologies must be able to resolve individual proteins in a manner that allows them to be uniquely identified, connected to their functional complexes, and associated with clinical outcomes.

The field of proteomics has recently progressed to very sophisticated and sensitive methods of protein representation. “Protein representation”, in this paper, includes peptides, and biochemical modifications such as the addition of lipid/carbohydrate moieties to proteins, and fragments and breakdown products. It also includes isoforms of the protein. Some proteomic technologies are so sensitive that they can detect millions of “peaks” within a small region of the spectra. The technical sophistication of these methods requires that we are much more careful how we use them and their exquisite sensitivity requires that we be more careful how we select our study population (e.g., age, gender, food intake, comorbidities), how we operationalize our data collection, handling and processing, and how we perform our data analysis and interpretation. Because of the large number of proteins that are usually assessed in a single experiment, issues of sample size and power must be properly addressed prior to beginning the experiment.

Some investigators believe that the data generated by a proteomic representation device, for example, spectra, are something real, that we are like looking at the proteins when we look at peaks. This would be an incorrect view of proteomics. Scientific instruments allow us to observe what we cannot detect directly by our senses by amplifying the phenomena. The analogy with a microscope is that if the lens is of poor quality or if it lacks sufficient resolution, what we see will be indistinct, distorted, and either not useful or misleading. Proteomic devices transform the underlying proteins, they provide a “representation” of the proteins; they do not show us the proteins themselves. One might suggest that this is a distinction without a difference, that the representation is so good that it is as if we were looking the protein itself. But the output of a proteomic device is not an exact copy of the protein; rather, it is a number that is the result of a series of manipulations and transformations of the raw data. Each manipulation and transformation has the potential to change the information, so that it no longer means what it originally meant in the sera. We are interested in devices that are “truth preserving”, i.e., that transmit as close to the true phenomena as is necessary for the success of the experiment. Proteomic representation devices are not *a priori* truth preserving, they must be proven to be so. In other words, before we can use proteomic representations in clinical medicine they must be proven to be an accurate characterization of the proteins they purport to represent.

For many diseases we do not possess tissue that can be analyzed, all we have are bodily fluids, e.g., urine, secretions, and sera. Sera is an inviting target for proteomics because the technology can represent circulating proteins concentrations. The down side of this approach is that the number of circulating proteins is unknown, many proteins circulate in bound form, and protein degradation products also circulate in the sera. In addition, one does not have tissue specificity – the circulating proteins represent the state of the entire body rather than the state of a disease. Because few proteins are unique to a specific tissue, proteins are expressed by multiple tissues; the linking of a specific protein concentration to a specific tissue/organ abnormality can be problematic. Further, disease and patient protein heterogeneity are difficult to define independently of the circulating proteins themselves.

In terms of their analysis, gene microarrays and protein spectra are very similar, and some investigators have applied the same methods to both, for example, hierarchal clustering. (Eisen et al., 1998; Perou et al., 1999) The view that gene microarrays and protein spectra are similar may arise from the fact that both consist of many variables at the molecular level of analysis. But genes and proteins are completely different phenomena, both in structure and function. Further, there are currently problems with the analysis of gene arrays, for example, the inability of investigators to replicate their work in even simple human systems (Catherino et al., 2003; Tsibris et al., 2002; Whang et al., 2003) or the work of others (Bullinger et al., 2004; Valk et al., 2004), that suggests that an *in toto* movement of methods from genes to proteins may not be advisable at this time and that investigators should be vary careful when creating a close analysis analogy between these two domains.

## Study Design

There are two types of studies, exploratory/discovery and validation. Exploratory/discovery studies do not test hypotheses, they do not perform power calculations, and the study design does not follow pre-specified rules. These studies may be used to find proteins and protein patterns that become the targets of validation research. Most investigators take great joy in publishing exploratory/discovery research and literature is replete with this work. The problem is that with enough bending and twisting of the methods and data almost any effect can look good. Most exploratory/discovery studies possess true phenomena but there is no way, based on what is published, to know if they are true. An approach based on self-restraint should be adopted and publication should wait until the appropriate phase in the validation process.

Once an investigator possesses a hypothesis, based on exploratory studies or biological plausibility, a study population can be defined, a study method, for example, case-control or cohort, can be established, and power calculations can be performed based on the hypothesized effect size and the variance of the variables. Power calculations in proteomics are rarely preformed and the studies are almost always underpowered, which usually results in the parameter estimates having too high a variance to be reliable.

There are many types of study designs including: pedigree (a kind of case report), retrospective case-control and cohort studies, prospective case-control and cohort studies, and randomized prospective cohort studies (usually called randomized controlled trials). Each of these types of studies has strengths and weaknesses that must be considered when designing a proteomics study. The main problem with nonrandomized studies is that there can be unmeasured covariates that were powerful predictors of outcome that were used in the patient selection process but that were not adjusted for in the analysis because they were not measured. Case-control studies are especially sensitive to patient selection bias, but any cohort study, including randomized studies, can possess patient selection bias, the difference is that in randomized studies, rather than affecting the results, the bias affects the generalizablility of the study, i.e., will others receive the degree of benefit demonstrated in the study. Further, because randomized studies usually use very specific entry criteria, to create a homogeneous study population, they are very sensitive to study design differences. This is why two large randomized studies with the same hypothesis can produce different results.

## Three phases of validation of a proteomic biomarker and biomarker patterns

*To validate a biomarker one must demonstrate, for a particular clinical use, that the biomarker reliably and accurately predicts a clearly defined clinical outcome in a distinct target population over an specified interval of time.* A properly validated biomarker will be reproducible across laboratories, investigators, and similar patient populations and it will maintain its predictive accuracy when used in community settings.

The process of biomarker validation is distinct from biomarker discovery. Once a putative biomarker has been discovered, it should be assessed through a formal validation process in order to determine if it is a reliable, accurate biomarker, and that it is clinically useful for a specific clinical outcome in a target population over a specified time interval. There are three phases of the validation of biomarkers, namely, analysis/testing, replication, and validation.

### Phase I

The first phase involves the analysis and testing of the biomarker by an investigator using a retrospective patient cohort in the investigator’s laboratory. At the start, the retrospective dataset should be split, usually 70/30, with 70% of the data being used for the analysis and 30% used to test the analysis. In this phase, the investigator is learning about the molecular biomarker and testing what has been learned. The molecular biomarker can be analyzed using as many laboratory methods, statistical techniques, thresholds (cut-off points), patient populations, independent variables, and outcomes as is required for the investigator to optimize its clinical utility. The effects of confounders can be determined and subgroups can be assessed. But, the optimized molecular biomarker should be assessed on the test dataset only one time. If more testing is necessary, then an additional dataset is needed.

If a biomarker is to be thresholded, there should be only one threshold, resulting in all patients being either positive or negative. The biomarker should not be stratified into three or more states, usually through the use of two or more thresholds. Further, the biomarker should not be divided into ranges, for example, quartiles, and the highest and lowest compared, rather, the entire range of the biomarker should be assessed for predictive accuracy because extreme cases are usually the most easily predicted. It is the middle range of outcomes, i.e., those patients who are not destined to die quickly and who are not at an extremely low risk of death, that are the most difficult to predict. It is the application of a biomarker to the middle of this population that reduces the its overall predictive accuracy. Therefore, if the investigator does not wish to make a single positive-negative threshold the biomarker should be left as a continuous variable.

The point of the first phase is to determine if the biomarker is sufficiently accurate to warrant its proceeding to the next phase of validation. Although there are no hard and fast rules regarding the accuracy of the molecular biomarker, other than the fact that it will decline at each phase of the validation process, the investigator should set a minimum test dataset ROC for each phase of the process, the threshold selected will depend on the costs and benefits inherent in the use of the biomarker.

Phase I studies, where the investigator has changed the analysis many times based on looking at the test results in order to improve the marker’s accuracy, do not merit publication. If the investigator only used the test dataset once, then those test results may be publishable. However, the fact that it is a Phase I biomarker study must be prominently stated in the introduction, methods and discussion sections. The publication of biomarker results requires the presentation of specific information including the exact laboratory method to be used to determine the value of the molecular biomarker, its threshold (cut-off), the target population, the clinical use of the biomarker, the outcome of interest, and the time interval of the outcome prediction. The specification of the study characteristics should be sufficiently detailed so that the independent investigator in another laboratory can independently assess the molecular biomarker’s predictive accuracy in his or her retrospective patient cohort.

### Phase II

The second phase is the replication of the testing component of the Phase I study by an independent investigator in another laboratory using a different retrospective patient population. A Phase II study begins with the original investigator specifying the validation characteristics of the molecular biomarker, usually in a publication. These characteristics are derived from the testing component of Phase I and can no longer be changed for Phase II since it is assumed that, based on the Phase I analysis and testing, these previously determined and optimized characteristics will provide the optimal predictive accuracy for the molecular biomarker. Phase II studies are publishable with the caveat that they should be identified as Phase II studies, and should include an explicit discussion of all the inherent limitations of a Phase II study.

### Phase III

The third phase is a prospective, multi-investigator, multi-institutional study. Prospective studies usually are very long, large, and expensive. Thus, there should be strong evidence from Phase II in support of the molecular biomarker if it is to progress to this phase. The molecular biomarker must be evaluated in Phase III using the same validation characteristics as were specified in the testing component of Phase I and assessed in Phase II.

To validate a proteomic biomarker one must demonstrate that it accurately predicts the outcome it purports to predict in its target population. The standard to which a valid biomarker is held is that other independent investigators are able to reproduce its predictive accuracy using independent, but similar populations.

For the purposes of this discussion we will use the term “biomarker” to refer to both an individual predictive factor and a group of factors (i.e., a proteomic pattern). It should be noted that we do not need to know a protein’s functions in order to use it as a predictive factor, but we should be able to identify it by name it if we are to reduce the number of nonplausible results. If we do not know its name, then we must be more methodologically rigorous in order to minimize error.

There are three phases to the validation of a biomarker, namely, learning, analysis/testing, and replication. Investigators, including this author, have used the nomenclature “learning training”, “learning test”, and “replication”. The nomenclature here differs from what has been used in the past because the approach proposed here is more rigorous than past validation approaches.

Each phase requires a dataset. The learning and analysis/testing datasets may be either independent datasets or they may derived from a larger dataset by randomly dividing the larger dataset into two subdatasets. The two subdatasets do no have to be of the same size. The replication dataset must be independent of both the learning and testing datasets. Although it would be ideal for all three phases to use prospectively collected data, the learning and testing datasets may be retrospective, but the replication dataset should be a prospectively collected dataset so that it can contain all the relevant factors, defined correct, and collected in the appropriate population.

In the learning phase the investigator selects one or more proteins or protein patterns to be candidate biomarkers. The learning dataset is then randomly split into a training and hold-out dataset, where the hold-out dataset is usually smaller than the training dataset. The biomarker is modeled using a statistical method and its accuracy is tested on the hold-out dataset. During the learning phase the investigator may assess many statistical methods, add or remove biomarkers, or modify the analysis in any way. There are no limitations on what may be done during this phase of the analysis. No accuracy assessment from the learning phase should be reported.

In the analysis/testing phase the investigator uses the trained model, the model that was developed in the learning phase, one time on the tests dataset. The test dataset had its outcomes removed. The biomarker of the test patients is run through the model, with the output of the model being an outcome prediction for each patient. The predicted outcomes are compared to the true outcomes and that predictive accuracy is reported. But it should be understood that the biomarker has not yet been validated. This is because the same investigator using the same dataset performed both the learning and the training. Many unknown and even unanticipated sources of bias could exist that could have affected the model and its predictions. Unfortunately, some investigators use the learning dataset to create many models and they test each model on the test dataset and they report the model with the highest accuracy. This is not an acceptable approach to the validation of a biomarker.

The hallmark of science is replication. For a biomarker to be valid it must be applied by an independent investigator on a prospectively collected, independent dataset. The process is similar to what occurs in the testing phase. The trained model is applied one time to the replication dataset. The predictions of the model are compared to the true outcomes and that predictive accuracy is the accuracy for the biomarker for that population. When reporting validation results, the parameter estimates and their variances should be presented and the raw data, with appropriate safeguards, should be made available to other investigators so they can assess the results.

The outcome that a biomarker predicts can be of any type. When there are more than two discrete outcomes, or when the outcome is continuous, it is usually not appropriate to compare the two outcome extremes, for example, the highest and lowest quartile. This is because it is usually the middle group that is the most difficult to predict, and which decreases overall predictive accuracy, because extreme cases are usually the most easily predicted.

Finally, one cannot perform serial comparisons, as a kind of robustness measure, to validate a biomarker unless an exact match is required. What this means is that one cannot take a set of biomarkers, i.e., a “pattern” (which implies a single unit), from the test phase and compare them to results from other datasets and claim that because some of the biomarkers from the other database match your biomarkers that those biomarkers that matched are validated. We cannot say this because of the inherent variability in proteomic biomarkers. Given long enough lists, some biomarkers will always match by chance. If the investigator wishes to compare for an exact match, where all the biomarkers in both lists have to match exactly, that would be an acceptable replication experiment. Some might find this too stringent a criteria, that an approximate match will do. An approximate match will result in the acceptance of many false patterns.

## Assessing biomarker accuracy

There are three components to biomarker accuracy, namely, the biomarker itself, i.e., the predictive power of the biomarker for the outcome of interest in the relevant population, the efficiency of the statistical method used to associate the biomarker with the outcome, i.e., how good the statistical model is at associating the independent-dependent variable pairs, and the method for assessing the accuracy of the model’s predictions, i.e., the method we use to measure the association between the model’s predictions and the true outcomes.

An important consideration in model building is model instability. Model instability occurs when the independent variables’ parameter estimates can vary widely, so that if the order in which the patients were presented to the statistical method and the order in which the variables were presented to the statistical method were both changed, then the parameter estimates will significantly change because of the high variance in the parameter estimate. To avoid model instability we need at least 15 – 20 events per independent variable in the model. (We also need 15 – 20 events per independent variable in the test and validation datasets.) With this number of events we can “fix” the relationship between the independent variable and the outcome. Events are defined as the least frequent of the outcomes. Thus, for a binary outcome, e.g., alive and dead, whichever event occurs least often is the event rate. The optimal ratio for assessing a binary outcome is a 50% event rate. As the event rate moves away from 50%, toward 0%, it becomes easier to make predictions because one can simply predict that the more frequent event will happen. For example, in terms of percent correct, if the event rate is 10% then one could be correct 90% of the time by always predicting the occurrence of the not-event.

This example illustrates why one cannot use percent correct as the measure of predictive accuracy. Statistical models can learn to “bet the frequency” and ignore the independent variables. In fact, with low event rates, rarely will any independent variables do as well as betting the frequency.

Another measure of predictive accuracy is sensitivity and specificity. This approach requires that the variables or the model output be made binary, i.e., that some threshold is applied to the variable or to the model output, for example, above a certain number is positive and below is negative. One problem with this approach is that thresholding variables usually reduces their accuracy. Another problem is that different investigators may pick different thresholds so that variables and models cannot be compared across investigators. Since comparison is necessary for validation sensitivity is not an appropriate accuracy measure.

Although proteomic investigators have reported 100% sensitivity and 95% specificity, (Petricoin, 2002) no biomarker for an important medical problem can achieve this degree of accuracy, if only because there is always variance, resulting in prediction error, in real-world classification tasks. In addition, there can be a bias in the study population, in the sample collection, handling, and storage, or in the processing of the samples that will affect predictive accuracy. Finally, one way to achieve high predictive accuracies is to select an easy task.

Although there are new methods under development, currently the area under the receiver operating characteristic curve (ROC) is the best measure of predictive accuracy. (Swets, 1996) It can be used to assess and compare the adequacy of statistical models. The ROC can be directly calculated by Somer’s D (Somer, 1962) or it can be approximated by its trapezoidal area. (Bamber, 1975) The area under the curve is a nonparametric measure of discrimination. The receiver operating characteristic area is independent of both the prior probability of each outcome and the threshold cutoff for categorization. Its computation requires only that the prediction method produce an ordinal-scaled relative predictive score. In terms of mortality, the receiver operating characteristic area estimates the probability that the prediction method will assign a higher mortality score to the patient who died than to the patient who lived. The receiver operating characteristic area varies from zero to one. When the predictions are unrelated to survival, the score is 0.5, indicating chance accuracy (flipping a coin). The farther the score is from 0.5 in either direction, the greater the accuracy, i.e., the better the prediction method is, on average, at predicting which of two patients with different outcomes will be alive. The ROC takes into account event rates below 50% so that “betting the frequency” results in a 0.5, chance, accuracy. One can think of the ROC as an estimate of accuracy across all possible sensitivity/specificity pairs. Significant differences in the receiver operating characteristic areas between two models can be tested following Hanley and McNeil (1982), or by calculating their asymptotic variances (algorithm available by email from the author), or by calculating the empirical variance using the bootstrap method. (Ffron, 1993) Although, in theory, the ROC is linear across its range, for most clinical problems the ROC is nonlinear in that it is more difficult as one moves from 0.5 to 0.6 to 0.7 to 0.8 to 0.9 to 1.0. This is because once one has predicted the easy cases, the remaining cases are harder to predict. Today, most ROC values reported in medicine are in the 0.70 – 0.80 range. Proteomics may allow researchers to discover biomarkers that achieve ROCs in the range of 0.8 – 0.9.

## Data analysis

Fundamental to the analysis of proteomic data is the fact that proteins are nonlinear and interactional. They are nonlinear because they are affected by enzymes and other proteins, and they are interactional because they are usually only active when complexed with other proteins. Therefore, any method for analyzing proteins must be able to capture nonlinearity and interactions. Most regression methods can deal with both by adding nonlinearity terms to each variable and adding all possible interactions between variables to the model. The problem is that as the number of variables increases the number of interactions increases exponentially and that the model quickly becomes overparameterized and unstable. Artificial neural networks with weight decay (Burke et al., 1995; Burke, 1996a, 1996b, 1998a, 1998b, Bostwick and Burke, 2001) and some other advanced statistical methods can effectively capture nonlinearity and interactions.

Another issue that must be addressed is that of integrating demographic level factors, e.g., age, gender, race, with clinical variables, e.g., tissue and serum factors, with genomic factors, with the proteomic factors. (Burke and Henson, 1999) A model that is used to assess proteomic factors, from spectra or any other source, should include these other factors with the proteomic factors and the proteomic factors should independently and significantly add information. There are substantive issues that arise when we do this. One important issue is that of cross-level colinearity, of which there is a paucity of examples because it has not, as yet, a well known problem. What this means is that in hierarchal systems the units at the lower level are constitutive of the units at the next higher level so when units at different levels are combined, there can be colinearity. In addition, when proteins interact with other proteins, if not properly analyzed, one of the proteins may be inappropriately removed because of colinearity.

Proteomics may be used to explore the mechanisms that give rise to a disease and they may be used, without knowledge of their role in the disease process, as predictive factors and thus allow the determination of either risk of disease, or existence of disease, or prognosis and treatment. (Burke, 1999) On a practical level pharmacoproteomics is the search for the proteomic factors that predict the disease’s response to a specific treatment. In other words, pharmacoproteomics is the discovery of proteomic patterns that are therapy-specific prognostic factors, i.e., that predict whether an individual patient with a particular disease will respond to a specific treatment and they may assume a dual role, also being the targets of therapy.

## Information theoretic perspective and massively parallel information

A large scale (thousands of proteins) protein spectrum creates an analog representation of the average relative quantity of a protein across a huge number of cells for a large number of proteins at a biologic moment in time for a particular patient. The output of a protein spectra contains the signal, the true average relative quantity of each protein, and noise, the spurious and background activity level. Protein spectra are a source of massively parallel information. They are able to provide a large amount of information in a non-serial manner (the information is not the result of a conditional sequence of investigative events). There are few examples of massively parallel information in science, thus there is little known about its analysis.

Initially it should be assumed that every data element in a massively parallel information representation has the potential to be a meaningful, i.e., to be a necessary but not sufficient part of the pattern. The reason for this assumption is that if it were not possible for each data element to be meaningful then massively parallel information would not necessary. It is precisely because any element could be important that we are interested in, and willing to deal with, the problems of massively parallel information. Typically microarray data contains thousands of expression measures per case and few cases. (Lander, 1999) Microarrays present an analytic problem that is NP-hard. NP stands for “non-deterministic polynomial time” and represents a class of computational tasks for which a potential solution can be checked efficiently for correctness, yet finding such a solution appears to require exponential time in the worst case.

Magnetic resonance imaging (MRI) might be relevant to protein spectra because it generates large amounts of information in parallel in an image processing paradigm. But its task is made orders of magnitude simpler by image processing having a preexisting natural distance metric, i.e., spatial proximity. In the protein spectra domain this would be similar to proteins being *a priori* arranged in the spectra in the natural order of their decreasing relatedness to a particular biologic function.

It is reasonable to ask why we should deal with the problems inherent in massively parallel information. Simply put massively parallel information is essential to understanding complex diseases. Because protein spectra represent information in a massively parallel fashion they can simultaneously present multiple components of a complex system. Prior to protein spectra proteomic research had to proceed in a stepwise fashion, from one biologic component to the next. While this is appropriate (but not optimal) for normal systems it is not a viable approach when dealing with complex (nonlinear, interactional) tightly integrated dynamical systems. Such systems must be examined and understood as a whole rather than as a series of pieces.

Three characteristics of a complex dynamical systems are: (1) they possess alternative pathways, i.e., there are different ways of accomplishing the same result, (2) each component has multiple functions, i.e., different activities that it can perform, and (3) pathways and functions are determined by the activities of other components of the system. This type of system cannot be successfully studied in pieces because when the study is completed the pieces will not fit together. In other words, disease is not a jigsaw puzzle whose pieces can be taken apart and put back together. (Burke, 2000)

## Protein patterns

“Pattern” can be operationally defined as a set of elements that occur in a systematic and meaningful-for-the-task manner. In the context of protein spectra, there are two types of pattern tasks. One type, pattern discovery, is the detection, or more correctly, the learning of a new pattern from the data. The other type, pattern recognition, is the recognition of a pattern when it occurs again, i.e., the ability to identify a pattern as an instance of a known pattern. Pattern discovery and recognition are ancient problems; their literature extends back to the ancient Greek philosophers. Today they are a central problem in the psychology of human perception. Pattern recognition is the less challenging problem because once a pattern is known templates and other approaches can be easily applied.

The analysis of large scale protein spectra-generated information is intractable when every protein is considered to be a continuous variable, when there are tens of thousands of proteins, when there is inter-patient variation, when there is disease variation (stage, subtype), when there is error in the proteomic technology, and when there are only a few exemplars of the pattern, i.e., only a few individuals are represented by a protein spectra. The analysis of large scale protein spectra can be simplified by thresholding each protein’s signal (creating a binary variable), minimizing disease and inter-patient variation, and increasing the number of patients. But even in this situation, because of protein modifications and other issues there are more proteins than one would like to analyze. In the simplest of conditions, when the proteins are considered binary variables, there are 2^n^ possible patterns (where n is the number of proteins and their variants). 2^n^ is still a very large number. Clearly, this is very high dimensional space. This space has its own characteristics, for example, the curse of multidimensionality. High dimensions space is extremely large and the data points move to the edges of the space.

## Two approaches to the analysis of proteomic data

One can approach the analysis of proteomic data from either a “top-down” or a “bottom-up” perspective. Top-down starts with all the data and reduces it based on selected heuristics, for example, the largest relative peaks (differential expression), or the clustering of relative peaks, or the principal components analysis of relative peaks. The bottom-up approach either assess all the m/z ratios individually in a exhaustive manner or tests the relationship between specific proteins in the spectra and the clinical outcomes, usually by hypothesis testing. The top-down approach is usually uses unsupervised learning methods, but it may use supervised learning in selected cases, and it is rarely able to use significance testing, whereas the bottom-up approach usually uses supervised learning methods and almost always uses significance testing.

## Sources of variance (error) in the analysis of proteomic data

There are several sources of error in the proteomic profile including: (1) The proteomic technology itself. Issues related to the various protein representation technologies will not be addressed in this paper; (2) the patients; (i) the type and composition of the specimen that is utilized, (ii) where in the disease process the patient is at specimen collection, and (iii) individual and population proteomic variation; (3) the disease; there may be sub-types of the disease and there may be different pathways that result in the final common pathway of the disease; (4) the collection, handling, and storage of the specimens; (5) the generation of the data from the specimens; (6) the inefficiency of the statistical method in capturing the pattern; (7) the measure of accuracy employed; (8) random error.

### Promising areas of investigation

There are several interesting approaches to protein spectra-based pattern discovery. Stochastic search methods may be useful for finding putative gene patterns. (George and McCulloch, 1993) These methods attempt to identify possible subsets of explanatory variables that can then be evaluated. Other approaches currently being assessed for their efficiency is dealing with massively parallel data include a combinatorial multivariate method (Califano, 2000) and mixture models. (Wallace and Dowe, 1997; Fraley and Raftery, 1998; Liu et al., 1999) The combinatorial method proposed by Califano is a supervised learning task that improves as the number of cases increases. Finite mixture models assume that the data arise from a mixture of several unknown heterogeneous populations. Titterington et al. (1985) point out that finite mixture distributions have been used as models since the work of Newcomb in 1886 and Pearson in 1894. Mixture models can be used for unsupervised and learning tasks. Many estimation methods can be applied to finite mixture problems including the method of moments, maximum likelihood, minimum chi-square, least squares, and Bayesian approaches. (Titterington et al., 1985; Roeder, 1994; Richardson and Green 1997; Bohning, 1999) Finally, artificial neural networks may be useful for the analysis of proteomic data. (Burke et al., 1995; Burke, 1996a, 1996b, 1998a, 1998b, Bostwick and Burke, 2001)

## Summary

Proteomics is a very difficult area of investigation because of the number of proteins in the human body, and because proteins, when they function, are nonlinear and interactional. Any proteomic technology that will be useful in this area must, eventually, be able to reliably and unambiguously distinguish individual proteins. Any analytic technology must be able to reliably and unambiguously relate individual proteins or groups of proteins to an outcome, Finally, all the research in this field must be replicated before it is used clinically. Any rush to publish without proper scientific rigor can mislead the field and potentially harm patients.

## References

[b1-cin-01-15] Bamber D (1975). The area above the ordinal dominance graph and the area below the receiver operating graph. J Math Psych.

[b2-cin-01-15] Bohning D (1999). Computer-Assisted Analysis of Mixtures and Applications.

[b3-cin-01-15] Bostwick DG, Burke HB (2001). Prediction of individual patient outcome in cancer: Comparison of artificial neural networks and Kaplan-Meier methods. Cancer.

[b4-cin-01-15] Bullinger L, Dohner K, Bair E (2004). Use of gene-expression profiling to identify prognostic subclasses in adult acute myeloid leukemia. New Engl J Med.

[b5-cin-01-15] Burke HB, Rosen DB, Goodman PH, Tesauro G, Touretzky DS, Leen TK (1995). Comparing the prediction accuracy of artificial neural networks and other statistical models for breast cancer survival. Advances in Neural Information Processing Systems.

[b6-cin-01-15] Burke HB, Fisher D, Lenz H (1996a). Statistical analysis of complex systems in biomedicine. Learning from Data: Artificial Intelligence and Statistics.

[b7-cin-01-15] Burke HB, Keller PE, Hashem S, Kangas LJ, Kouzes RT (1996b). The importance of artificial neural networks and biomedicine. Applications of Neural Networks in Environment, Energy, and Health.

[b8-cin-01-15] Burke HB, Hanausek M, Walaszek Z (1998a). Integrating multiple clinical tests to increase predictive accuracy. Methods in Molecular Biology:, Vol XX: Tumor Marker Protocols.

[b9-cin-01-15] Burke HB (1998b). Applying artificial neural networks to clinical medicine. J Clin Ligand.

[b10-cin-01-15] Burke HB, Henson DE (1999). Evaluating prognostic factors. CME J Gyn Onc.

[b11-cin-01-15] Burke HB (2000). Discovering clinically significant patterns in microarray generated data. Mol Diagnosis.

[b12-cin-01-15] Califano A (2000). SPLASH: Structural pattern localization algorithm by sequence histograming. Bioinformatics.

[b13-cin-01-15] Catherino WH, Prupas C, Tsibris JCM (2003). Strategy for elucidating differentially expressed genes in leiomyomata identified by microarray technology. Fertil Steril.

[b14-cin-01-15] Efron B, Tibshirani RJ (1993). An Introduction to the Bootstrap.

[b15-cin-01-15] Eisen MB, Spellman PT, Brown PO, Botstein D (1998). Cluster analysis and display of genome-wide expression patterns. Proc Natl Acad Sci.

[b16-cin-01-15] Fraley C, Raftery AE (1998). How many clusters? Which clustering method? – Answers via Model-Based Cluster Analysis. Computer J.

[b17-cin-01-15] George E, McCulloch R (1993). Variable selection via gibbs sampling. JASA.

[b18-cin-01-15] Hanley JA, McNeil BJ (1982). The meaning and use of the area under the receiver operating characteristic (ROC) curve. Radiology.

[b19-cin-01-15] Lander ES (1999). Array of hope. Nature Genetics.

[b20-cin-01-15] Liu JS, Neuwald AF, Lawrence CE (1999). Markovian structures in biological sequence alignment. JASA.

[b21-cin-01-15] Perou CM, Jeffrey SS, van de Rijn M (1999). Distinctive gene expresión pattersn in human mammary epithelial cells and breast cancers. Proc Natl Acad Sci.

[b22-cin-01-15] Petricoin EF, Ardekani AM, Hitt BA, Levine PJ, Fusaro VA, Steinberg SM, Mills GB, Simone C, Fishman DA, Kohn EC, Liotta LA (2002). Use of proteomic patterns in serum to identify ovarian cancer. Lancet.

[b23-cin-01-15] Richardson S, Green PJ (1997). On Bayesian analysis of mixtures with an unknown number of components. JRSSB.

[b24-cin-01-15] Roeder K (1994). A graphical technique for determining the number of components in a mixture of normals. JASA.

[b25-cin-01-15] Somer RH (1962). A new asymmetric measure of association for ordinal variables. Am Sociological Rev.

[b26-cin-01-15] Swets JA (1996). Signal Detection Theory and ROC Analysis in Psychology and Diagnostics: Collected Papers.

[b27-cin-01-15] Titterington DM, Smith AFM, Makov UE (1985). Statistical Analysis of Finite Mixture Distributions.

[b28-cin-01-15] Tsibris JCM, Segars J, Coppola D (2002). Insights from gene arrays on the development and growth regulation of uterine leiomyomata. Fertil Steril.

[b29-cin-01-15] Valk PJM, Verhaak RGW, Deijen MA (2004). Prognostically useful gene-expression profiles in acute myeloid leukemia. New Engl J Med.

[b30-cin-01-15] Wallace CS, Dowe DL MML mixture modelling of multi-state, Poisson, von Mises circular and Gaussian distributions.

[b31-cin-01-15] Wang H, Mahadevappa M, Yamamoto K, Wen Y, Chen B, Warrington JA, Polan ML (2003). Distinctive proliferative phase difference in gene expression in human myometrium and leiomyomata. Fertil Steril.

